# Multimodal Breathing Control: Pontomedullary Mechanisms and Current Perspectives

**DOI:** 10.1002/bies.70072

**Published:** 2025-09-22

**Authors:** Nathan A. Baertsch, Elora Reily, Jonathan Sedano, Ryan S. Phillips, Joseph W. Arthurs

**Affiliations:** ^1^ Center For Integrative Brain Research Seattle Children's Research Institute Seattle USA; ^2^ Department of Pediatrics University of Washington Seattle USA; ^3^ Center For Excellence in Neurobiology of Addiction, Pain, and Emotion University of Washington Seattle USA

## Abstract

Breathing is a vital, continuous behavior that maintains physiological homeostasis, yet it is also remarkably flexible—modulated by volitional, emotional, and behavioral states. This review highlights recent advances in understanding how distributed neural circuits, particularly in the ventrolateral medulla and dorsolateral pons, integrate both homeostatic and non‐homeostatic influences on respiratory control. We examine how higher‐order brain regions interact with brainstem rhythm generators such as the preBötzinger complex, emphasizing a dynamic, state‐dependent framework for respiratory regulation. Once considered a reflexive brainstem function, breathing is now recognized as the emergent output of interconnected networks that flexibly adapt rhythm and pattern based on internal state, behavior, and environmental context. Grasping this complexity is critical for understanding both the normal versatility and pathological vulnerability of respiratory control.

## Graphical abstract

Breathing is typically automatic, ensuring homeostasis, but it is also shaped by emotion, behavior, and volition. This review synthesizes our current understanding of the flexible neural mechanisms that enable multimodal control of breathing, emphasizing the role of pontomedullary circuits.

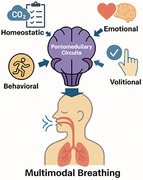

## 1 Introduction

Breathing is essential for mammalian life, maintaining homeostasis through rhythmic air exchange. This requirement is constant—breathing must continue from birth until death—but not unchanging since it must adapt to fluctuating metabolic or environmental demands. Uniquely among vital functions, breathing is also subject to volitional control and profoundly reshaped by behavioral and emotional states unrelated to homeostatic demands. This review focuses on recent advances in pontomedullary mechanisms of multimodal respiratory control, examined at multiple levels—from rhythm‐generating neurons in the preBötzinger complex (preBötC), to distributed population dynamics across the ventral respiratory column (VRC), to the integration of higher‐order influences by the dorsolateral pons. We explore how cellular properties and local circuit dynamics enable flexible rhythmogenesis, how coordinated VRC activity supports robust yet reconfigurable breathing patterns, and how pontine circuits coordinate homeostatic regulation with behavioral and emotional modulation. Special emphasis is placed on the parabrachial nucleus (PBN), highlighting its molecularly and developmentally defined subpopulations and their roles in shaping distinct breathing modes. Along the way, we identify key questions and experimental directions that will advance our understanding of the neural mechanisms that govern respiratory control and how these diverse layers of regulation interact to produce dynamic multimodal breathing.

## 2 Defining Relationships Between Different Types of Breathing Control

**FIGURE 1:**
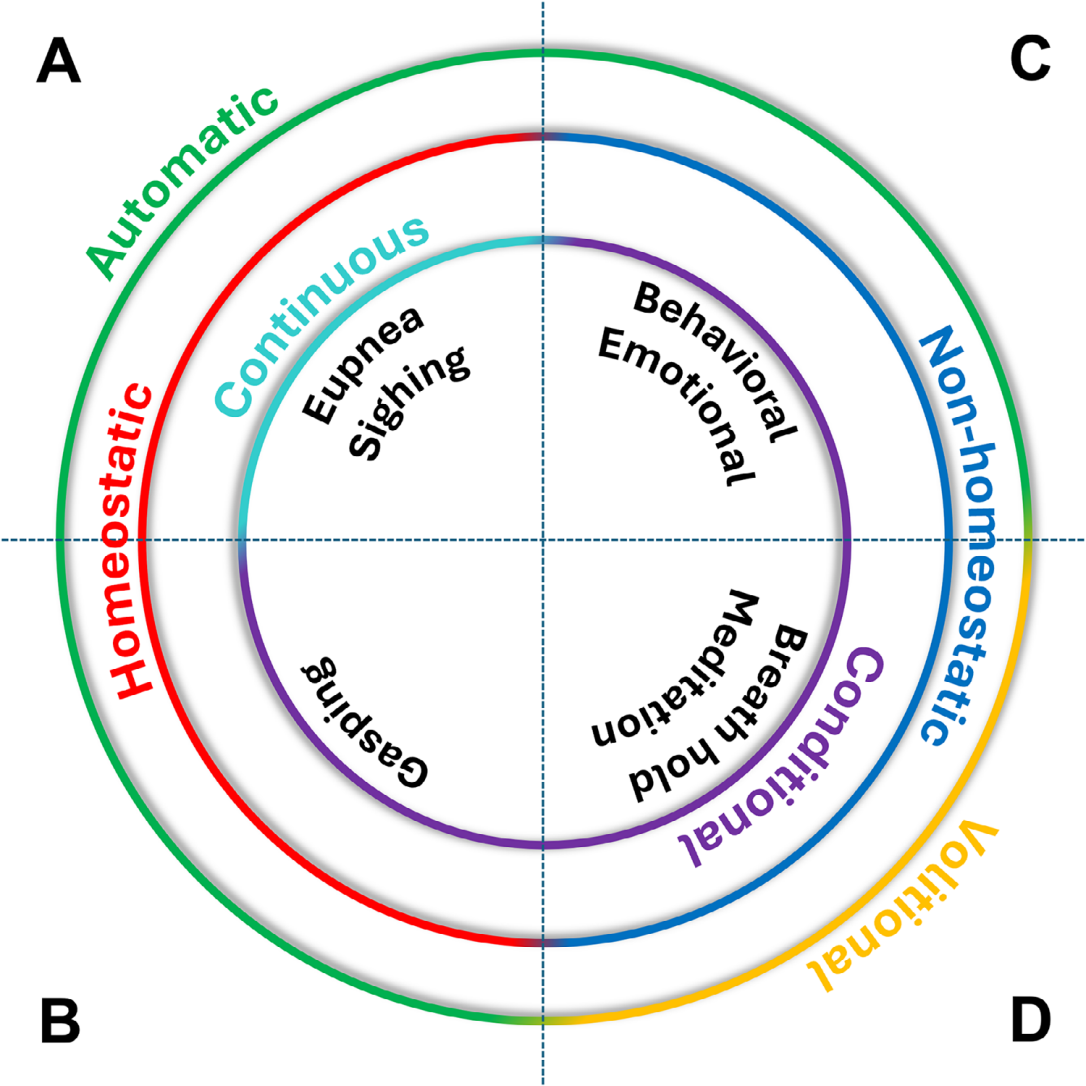
Terminology of multimodal breathing control.

In healthy individuals, breathing generally occurs with ease and goes unnoticed. Most of the time, it is automatic—requiring no conscious thought—and when respiratory effort aligns with sensory feedback, there is remarkably little awareness of breathing. This can lead to the presumption that its underlying neuronal control is simple. Yet, when breathing becomes pathological—as in sleep‐disordered breathing, panic disorders, and neurodegenerative disease—it can no longer be taken for granted, and its underlying complexity becomes readily apparent. Breathing is governed by multiple types of modulatory control, driven by distinct yet integrated neural mechanisms. Here, we offer a framework for conceptualizing the relationships between different modes of breathing control (Figure 1).

Automatic control reflects the vital homeostatic functions of breathing that ensure the continuous regulation of blood gases and pH (Figure 1A). This includes regular tidal breathing known as eupnea and its reflexive modulation by interoceptive sensory feedback, as well as deeper inspirations that occur periodically, known as sighs, which ensure alveoli remain open and gas‐exchange is efficient [1, 2]. Although the rate of eupnea and sighing are influenced by many factors, they occur continuously throughout life. Breathing control can also be conditionally transformed to protect homeostasis (Figure 1B). For instance, during severe hypoxia or asphyxia, intense inspirations with prolonged pauses—gasps—occur as a last‐ditch autoresuscitative effort to restore homeostasis. These automatic forms of breathing (Figure 1A,B) persist across sleep‐wake states and even under anesthesia, ensuring homeostasis is uninterrupted.

Breathing is also automatically modulated for purposes unrelated to homeostasis. These automatic, non‐homeostatic mechanisms (Figure 1C) seamlessly and, often imperceptibly, integrate breathing with conditional behaviors or emotions such as vocalization, coughing, swallowing, sniffing, laughing, crying, excitement, fear, and relaxation [3, 4]. One may spend hours chatting and laughing with a friend without any awareness that to do this, their eupneic breathing pattern has been profoundly disrupted. Inspiration becomes quick and deep, preparing enough air for the subsequent phrase or sentence, while exhalation slows and tightens to shape syllables. Laughter disrupts eupnea further, forcefully expelling air from the lungs in abrupt and irregular patterns. All the while, breathing maintains its basic homeostatic function, exemplifying the remarkable ability of breathing to be automatically integrated with other volitional or reflexive behaviors that utilize the same orofacial structures.

This level of automaticity is unique among somatic motor systems, which usually rely on volitional control and are strongly gated by neuronal state. For example, locomotion requires wakefulness, conscious initiation, and active coordination with other motor behaviors. A hurdler must choose when to run, how fast, and plan when to jump to clear the obstacles without calamity. Overriding its underlying automaticity, breathing can be conditionally subjected to similar volitional control (Figure 1D). Meditation and yoga practice utilize the intentional control and awareness of breathing to influence physical and mental states. For a flutist, breath control is an art that requires substantial thought, ensuring timely inhalations and exhalations that purposefully manage airflow, air pressure, and timing with fine motor control. A child may hold their breath in protest; a freediver does so willfully, holding their breath for many minutes while swimming to great depths.

These forms of breath control require consciousness. When it is lost, automatic homeostatic regulation resumes—illustrating the state‐dependence and automaticity of non‐homeostatic and homeostatic respiratory control, respectively. The conflict between these systems is stark in freediving: CO_2_ builds during breath‐hold, triggering dyspnea. Eventually, falling O_2_ may induce blackout, leading to release of the volitional breath hold and resumption of homeostatic breathing control, likely in the form of gasps—a dire outcome if the diver remains submerged. Yet this tension is familiar: sprinting may leave one breathless while speaking, or laughter may make it hard to catch a breath. Similar conflicts arise in pathology. In psychological disorders such as panic attacks, emotional control of breathing competes with homeostatic drive, producing dyspnea [5]. Conversely, in conditions such as asthma, COPD, pneumonia, ALS, spinal cord injury, or sepsis, impaired homeostatic control may prompt compensatory volitional effort—leading to increased awareness of breathing, poor coordination, and dyspnea [6].

Most neuroscience research on the control of breathing has focused on its most vital functions. As a result, we have gained substantial insight into the neural mechanisms that support homeostatic respiratory control, despite remaining knowledge gaps. In comparison, the neural basis of non‐homeostatic breathing—its regulation, interaction with homeostatic systems, and role in respiratory pathologies—is far less understood.

## 3 Origins of Homeostatic Respiratory Rhythm and Pattern

The automatic properties of breathing, which ensure uninterrupted function, offer a distinct experimental advantage. Among rhythmic motor functions, breathing is uniquely preserved under anesthesia and even persists in ex vivo preparations that permit invasive and rigorous interrogation of its critical neuronal and molecular substrates. Nearly three centuries ago, it was recognized that removing the cerebral hemispheres did not stop breathing, whereas medullary lesions did [7]. Henceforth, increasingly refined lesion studies confirmed that the medulla contains a convergence point of interconnected nuclei that integrate vital respiratory and circulatory functions—the “noeud vital” [8]. During the 1960s and 1970s, the concept of the ventral respiratory group (VRG) emerged from pioneering extracellular recordings in decerebrate or anesthetized animal models, identifying respiratory‐modulated neurons distributed along the ventrolateral medulla [9, 10, 11]. At the time, the VRG was proposed as a central hub for respiratory control, containing distinct neuronal populations active during inspiration and expiration.

Later work demonstrated that medullary circuits alone can sustain rhythmic breathing, even in isolated brainstem—spinal cord preparations from neonatal rats [12]. In 1991, Smith and colleagues undertook a seminal effort to define the minimal medullary region sufficient to generate respiratory rhythm [13]. Serial sectioning of rhythmic brainstem‐spinal cord preparations revealed that a small bilaterally connected network that produces rhythmic inspiratory motor output could be captured in a ∼300 µm thick coronal slice. This region, dubbed the preBötzinger Complex (preBötC), is now considered the rhythmogenic core of the respiratory central pattern generator [14, 15, 16, 17].

To develop a mechanistic understanding of preBötC rhythmogenesis, researchers have relied heavily on these in vitro slice preparations. This approach has been invaluable not only for localizing the most critical site for respiratory rhythmogenesis, but for providing insights into the relevant cell‐types, membrane currents, synaptic properties, and neuromodulatory influences [18, 19, 20, 21, 22, 23, 24]. Much has been learned, yet even in the preBötC network in vitro, where the stereotypy of the rhythm implies a simple underlying mechanism, defining this mechanism has been difficult and a matter of longstanding debate [25, 26, 27, 28, 29].

## 4 The Illusion of Rhythmogenic Mechanism in the preBotzinger Complex

Inspired by the mindset that led to the discovery of the preBötC, efforts to define its rhythmogenic mechanism have been grounded in necessity and sufficiency criteria. Early observations identified a subset of preBötC interneurons that continued to burst rhythmically when synaptic interactions were blocked [30]. These intrinsically bursting or “pacemaker” neurons offered a parsimonious (Occam's razor) explanation: a specialized subpopulation with inherent rhythmicity drives network‐wide inspiratory bursts. Intrinsic bursting also suggested the involvement of a specialized membrane current [31], later identified as persistent sodium current (I_NaP_) [32]. However, I_NaP_ is not unique to pacemaker neurons, but ubiquitous in the preBötC [33]; and attempts to demonstrate the necessity or sufficiency of pacemakers for preBötC rhythmogenesis had many caveats [34].

Competing network‐based theories were developed that emphasized excitatory synaptic interactions over intrinsic membrane properties. The most recent version, known as “burstlet theory”, is based on observations that ramping spiking activity often precedes inspiratory bursts and, under some conditions, persists without them [35, 36]. These rhythmic changes in spiking activity that are subthreshold for inspiratory bursts—“burstlets”—are hypothesized to originate from increasingly synchronous excitatory synaptic interactions that do not depend on specialized intrinsic properties like I_NaP_ [37, 38].

To reconcile these competing views, recent work utilizing computer simulations of the preBötC has proposed a unifying framework for preBötC rhythmogenesis that integrates cellular and network‐level dynamics [39]. This model introduced a key insight: the shape of individual action potentials interacts with the voltage dependence of I_NaP_ to conditionally shape a neuron's firing mode. In model neurons with modifiable spike amplitude or afterhyperpolarization, small changes in spike shape can abolish or promote intrinsic bursting. When assembled into model networks, removal of intrinsic bursting did not stop network rhythmogenesis but shifted dynamics toward burstlet‐like activity with enhanced preinspiratory spiking. Conversely, in the same network, tuning spike shape to promote intrinsic bursting and eliminate pre‐inspiratory spiking also failed to preclude rhythmogenesis. Unlike these spike‐shape‐sensitive firing modes, I_NaP_ remained essential for preBötC rhythm generation across all spike shape configurations, disentangling the conflated roles of pacemakers and I_NaP_.

This framework redefines the spiking behaviors—bursting or preinspiratory ramping—of individual preBötC neurons, not as fixed or essential features, but as state‐dependent phenotypes that emerge from dynamic interactions between network activity and intrinsic properties like spike shape. The prevalence of these spiking phenotypes reflects conditional shifts in network configuration, rather than fundamental changes in rhythmogenic mechanism. This perspective helps reconcile the variability and apparent contradictions that have long challenged both the pacemaker and burstlet theories. Conceptually, it shifts the focus from identifying a singular, immutable mechanism to understanding how flexible cellular‐network interactions support rhythm generation under diverse conditions.

This unified view also offers a mechanistic foundation for the impressive flexibility of non‐homeostatic breathing under varied behavioral or emotional contexts. However, despite its conceptual appeal, the model remains grounded in in vitro data, and its applicability to breathing under naturalistic conditions in the intact system has yet to be rigorously tested. Key questions remain: What network state characterizes eupnea in vivo? What factors modulate spike shape and network dynamics under normal or pathological conditions? And how is the network reconfigured during transitions from homeostatic to non‐homeostatic breathing, or from continuous to conditional respiratory modes? Indeed, despite increasing appreciation that the brainstem contains many interacting circuits capable of producing rhythmicity in distinct ways [40, 41], it is generally assumed that modulation of a single preBötC rhythm‐generating mechanism supports all forms of breathing—from metabolic adaptation to emotional regulation. Yet optogenetic stimulation of preBötC neurons typically reproduces breathing patterns consistent with reflexive, homeostatic regulation [42, 43, 44, 45, 46, 47], not the rapid, flexible breathing patterns associated with behavioral or emotional control in awake mice [48, 49]—calling into question the mechanistic commonality underlying different forms of inspiration.

Addressing these gaps will require studies that test model predictions in the intact, behaving brain. The advent of large‐scale electrophysiology, genetically encoded sensors, and precise circuit manipulation and gene‐editing tools now makes it feasible to manipulate specific cellular or circuit features [50] and probe network state across physiological, behavioral, and emotional contexts. Ultimately, understanding respiratory rhythm generation may require moving beyond strict necessity/sufficiency criteria toward frameworks that embrace the emergent and degenerate nature of neural circuits [51]. Just as perception is shaped by the brain's tendency to favor simple, coherent interpretations (the law of Prägnanz), our understanding of rhythmogenesis must accommodate the possibility that breathing is generated not by a single, elegant solution, but by a flexible repertoire of interacting mechanisms optimized for robustness across conditions.

## 5 Integration Within the Ventral Respiratory Column

Inspiration forms the foundation of every mammalian breath. Yet each respiratory cycle may include additional phases of active motor output that are dynamically and conditionally assembled to shape the respiratory pattern [10, 16]. These occur during the period between inspirations, as air exits the lungs. The first, post‐inspiration, slows expiratory airflow and facilitates the integration of non‐ventilatory behaviors such as vocalization, coughing, and swallowing. The second, active expiration, forcefully expels air from the lungs when ventilatory demand is elevated, as during exercise [52].

Like the discovery of the preBötC, regions of the ventral medulla implicated in post‐inspiration and active expiration—namely, the postinspiratory complex (PiCo) and parafacial lateral region (pFL)—were defined using a similarly reductionist approach [53, 54]. Researchers identified neurons active during specific respiratory phases and used necessity and sufficiency criteria to map these regions. While this clarified the minimal circuitry capable of generating each phase, it may overlook emergent properties of the broader integrated network—the ventral respiratory column (VRC)—that underlie its ability to produce robust yet labile breathing. Rather than functionally isolated, rostrocaudally‐oriented compartments, recent evidence points to distributed and integrated VRC architecture where the breathing rhythm is shaped by widespread, interactive network dynamics.

Large‐scale electrophysiological recordings emphasize this distributed and dynamic nature of the VRC in vivo. High‐density recordings of >15 000 neurons within the ventrolateral medulla revealed that neurons with phase‐related activity patterns—pre‐inspiratory, inspiratory, post‐inspiratory, and expiratory—are not neatly confined to discrete anatomical compartments [55], nor do they form distinct phase‐related groups when clustered based on their spiking activity. Instead, their activity patterns form a continuous distribution across the respiratory cycle, following a low‐dimensional, rotational trajectory through neural state space. These findings suggest that respiratory phase transitions arise from coordinated VRC‐wide activity rather than sequential activation of anatomically distinct modules.

Complementary in vitro work has shown that the intact VRC produces a more stable and robust inspiratory rhythm than the isolated preBötC, suggesting adjacent regions bolster rhythmogenesis [56]. These studies also revealed a trade‐off between rhythm strength and flexibility: when inspiratory activity is especially strong, it expands rostrally beyond the preBötC core. This spatial expansion is modulated by sensory feedback—particularly from the vagus nerve—and the excitation‐inhibition balance, which enables tuning of respiratory network size to broaden the frequency range of breathing rhythms.

A striking example of this adaptability is the shift from eupnea to gasping. Under severe hypoxia or asphyxia—such as during airway occlusion—gasping emerges as a synchronized, all‐or‐none pattern that aids airway clearance and reoxygenation [57]. This transition involves a dramatic VRC reorganization: inhibition is suppressed, and inspiratory activity becomes dominant in regions rostral to the preBötC, while also expanding the portion of the VRC capable of inspiratory rhythmogenesis [55, 56, 58]. Such reconfiguration may explain why gasping may persist even after localized lesions of the preBötC abolish eupnea [59], which have challenged the idea of a discrete preBötC core network as the exclusive site of inspiratory rhythm generation.

Together, these findings reinforce the view of the VRC as an integrated and dynamically reconfigurable network, in which distributed neuronal populations interact to support both the stability of eupnea and the adaptability required for survival under stress. Just as rhythm generation in the preBötC arises from interdependent cellular‐network interactions rather than fixed neuronal features, broader respiratory patterning likely emerges from coordinated activity across multiple VRC regions. Gasping illustrates how such network‐level reorganization can support homeostatic responses. Whether similar reconfigurations support non‐homeostatic breathing patterns—shaped by emotion, volition, or behavior—remains a compelling open question. Addressing this will be essential to fully understand how VRC architecture enables complex, flexible respiratory patterns across physiological and behavioral states.

## 6 Bidirectional Integration of the VRC with the Dorsal Pons

A diverse constellation of neurons surrounding the brachium conjunctivum (superior cerebellar peduncle) in the dorsolateral pons—collectively referred to as the parabrachial nucleus (PBN)—communicates with nearly every major region of the central nervous system, from cortex to spinal cord [60, 61]. Some definitions of the PBN include the ventrolaterally adjacent Kölliker‐Fuse (KF) nucleus. The role of this region in respiratory control has been recognized for decades [62], yet its functions extend well beyond breathing. The PBN serves as a critical integrative hub for many behaviors, affective states, and autonomic processes [63, 64], complicating efforts to isolate its specific contributions to respiratory rhythm and pattern regulation. Most insights into pontine involvement in breathing stem from lesion studies, pharmacological manipulations, or broad perturbations [65, 66], leaving many interpretations inferential and key questions about how ponto‐medullary interactions regulate breathing unresolved.

Anatomical tracing has revealed robust bidirectional connections between the PBN and VRC [67, 68]. These reciprocal pathways likely mediate top‐down modulation and bottom‐up feedback, enabling dynamic tuning of respiratory rhythm and pattern. Glutamatergic projections from the KF to the VRC are believed to influence the timing and transitions between respiratory phases—particularly the termination of inspiration, often called the “inspiratory off‐switch” [69, 70]. Meanwhile, distinct subpopulations within the PBN appear to modulate breathing rate and depth in response to behavioral, emotional, or metabolic cues, such as arousal or threat [71, 72]. In turn, ascending feedback from the medulla likely conveys information about respiratory phase, frequency, or chemosensory signals, enabling pontine circuits to fine‐tune respiratory output [73]. With modern cell‐type and projection‐specific tools, the precise functional roles of these pathways can now be dissected in vivo.

**FIGURE 2:**
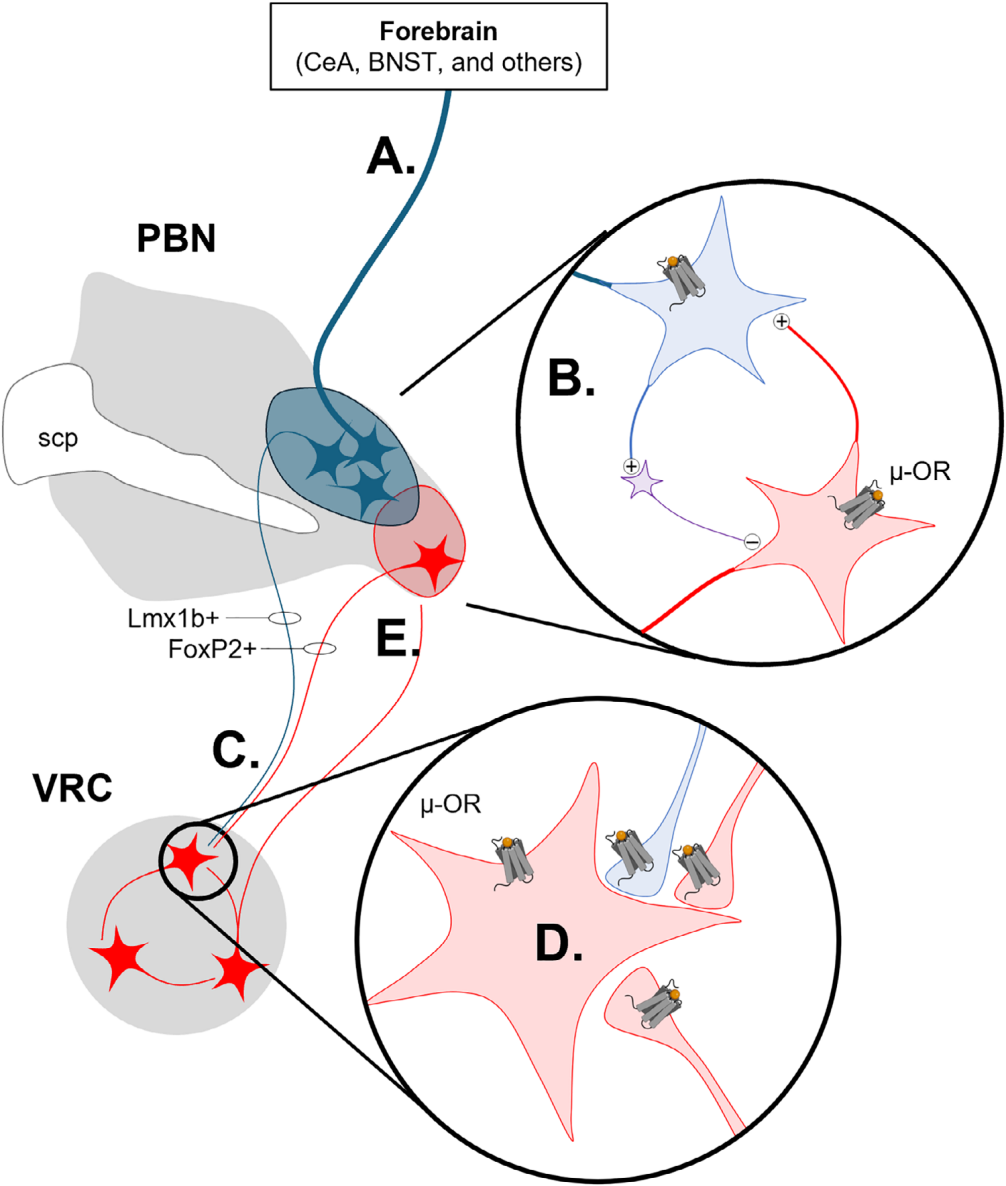
Working model of opioid effects on multimodal respiratory control. (A) Ascending Lmx1b+ PBN circuits are impaired, reducing breathing awareness (e.g., arousal to CO_2_ and dyspnea). (B) Impaired local PBN excitatory and inhibitory circuit interactions lead to uncoordinated homeostatic and non‐homeostatic breathing control. (C) Suppressed pre‐synaptic excitatory input to the VRC alters network state, making rhythmogenesis less robust. (D) VRC (preBötC) neurons are hyperpolarized, and recurrent excitatory interactions between them are suppressed, impairing rhythmogenesis. (E) Feedback from the VRC to the PBN is reduced, impairing timing and coordination.

Disruption of pontomedullary communication contributes to respiratory pathologies, most notably opioid‐induced respiratory depression (OIRD)—the underlying cause of nearly 200 opioid‐overdose deaths daily in the United States [74, 75]. Opioids directly suppress pontine and medullary neurons and weaken the excitatory synaptic interactions between them, disrupting local and long‐range connectivity within the respiratory network (Figure 2). The KF to the VRC pathway—a circuit believed to supply excitatory drive important for continuous respiratory rhythmogenesis—is particularly vulnerable to opioid inhibition [76]. Opioids impair this circuit at multiple levels: they inhibit KF neuron somata, reduce glutamate release at KF terminals in the VRC, and directly suppress postsynaptic VRC neurons via hyperpolarization and impaired local excitatory transmission [76, 77]. These cumulative effects dampen excitability of the pontomedullary respiratory network. In large‐scale electrophysiological recordings, this manifests as a marked slowing of VRC rotational population dynamics [55], accompanied by slow, shallow, irregular breathing that can culminate in terminal apnea.

This example underscores the important role of pontomedullary integration in maintaining stable homeostatic breathing and illustrates how network‐level dysfunction can destabilize respiratory control under pathological conditions. Yet these interactions are likely nuanced, context‐dependent, and dynamically regulated. Clarifying the specific circuits and cell types that mediate pontomedullary communication and how they operate alongside other top‐down pathways [68] will be key to understanding the rich repertoire of respiratory control mechanisms.

## 7 Unravelling Functionally Distinct Pontine Circuits for Breathing Control

Multiple features have been used in attempts to define functionally distinct subpopulations of PBN neurons, including developmental lineage, subregional anatomy, projection pattern, and transcriptional profile [71, 78, 79]. However, these classification schemes often do not align. For example, transcriptionally defined clusters do not neatly correspond to specific projection targets and are often distributed across PBN subregions [80], complicating efforts to determine which of the ∼40 000 PBN neurons [81] mediate specific functions.

**FIGURE 3:**
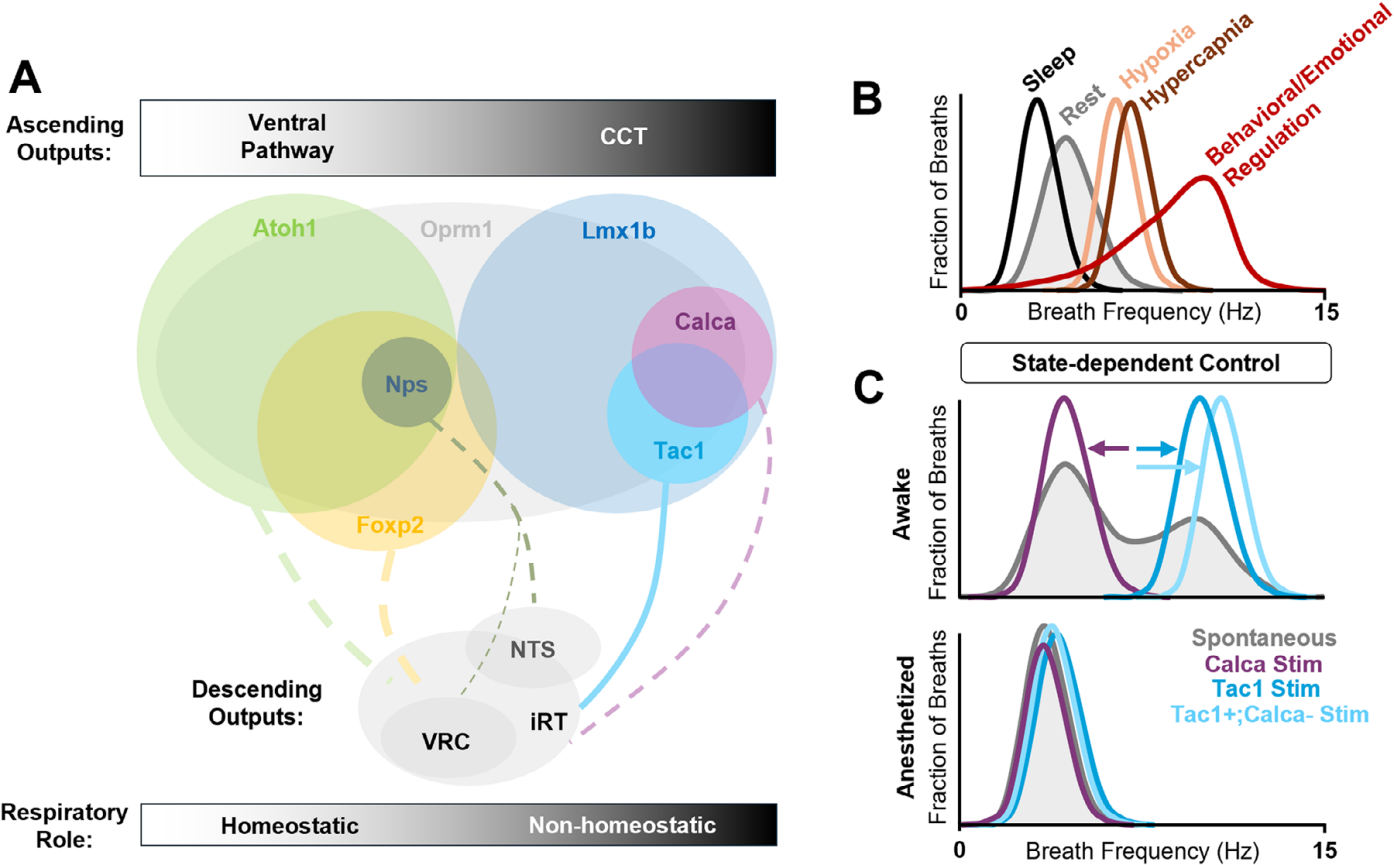
Relationships of PBN populations to breathing control. (A) Developmental lineages of the PBN and transcriptionally‐defined subpopulations with known respiratory functions. Dotted lines indicate projections that have been anatomically, but not functionally, defined. (B) Effects of changes in state on the distribution of breathing frequency. (C) State‐dependent effects of Calca and Tac1 PBN populations. In the awake state, rapid behaviorally‐driven breathing patterns are suppressed by activation of Calca neurons and driven by activation of Tac1 neurons. Effects of Tac1 and Calca neurons are lost under anesthesia. iRT, intermediate reticular zone.

The PBN consists primarily of two developmentally distinct macropopulations of glutamatergic neurons: one derived from Atoh1‐expressing progenitors and another defined by expression of the transcription factor Lmx1b (Figure 3). These populations are mutually exclusive but spatially intermingled across PBN subregions [81]. Atoh1‐derived neurons are more numerous and tend to localize rostrally, dorsolaterally, and medially, whereas Lmx1b neurons are skewed caudally and laterally, especially concentrated within the external lateral PB (PBel). These developmental lineages also favor—but are not limited to—different output pathways: Lmx1b PBel neurons project via the central tegmental tract (CTT) to forebrain targets such as the bed nucleus of the stria terminalis (BNST) and central amygdala (CeA), while Atoh1‐derived neurons tend to project via the “ventral pathway” to midbrain and hypothalamic regions [78, 81, 82]. Other outputs, including a periventricular route via the periaqueductal gray (PAG) and a descending pathway to the reticular medulla, including the VRC, arise from both lineages [78, 80]. These developmental origins shape adult gene expression [79], with excitatory PBN neurons divided into at least 13 clusters, primarily based on differential expression of neuropeptide and G‐protein‐coupled receptor (GPCR) genes [80]. While these markers—and the growing suite of Cre‐driver lines—facilitate functional dissection, overlapping expression can complicate interpretation. Some transcripts are highly enriched in specific clusters but not exclusive to them, while others are broadly expressed across multiple clusters. This layered heterogeneity underscores the challenges in defining functionally distinct PB circuits.

Studies using gene expression and projection targets are beginning to reveal specialized roles for PBN neuron subpopulations in homeostatic and non‐homeostatic breathing. Broadly, Atoh1‐derived populations appear to support stable, reflexive homeostatic breathing, while Lmx1b‐expressing populations modulate breathing in conditional, context‐dependent ways. Loss of Atoh1‐lineage PBN neurons destabilizes breathing and impairs CO_2_ responses [83], highlighting their essential role in homeostatic respiratory drive. Most Atoh1‐derived neurons express the transcription factor FoxP2, though a small subset lacking FoxP2 in the dorsolateral PBN [81] is enriched in prodynorphin (Pdyn)‐expression and implicated in thermoregulation [84] but not breathing (unpublished observations). While most FoxP2 neurons follow the ventral pathway [78], many innervate the VRC [83, 85]. Functionally, many FoxP2 neurons located in the central lateral (PBcl) and KF subnuclei are activated by CO_2_ exposure and transitions out of NREM sleep [86]. Optogenetic activation enhances respiratory rate, while silencing blunts the CO_2_ response but does not affect basal breathing [86]. However, FoxP2 is expressed across at least four transcriptionally distinct clusters of PBN neurons [80]. Neurons co‐expressing FoxP2 and Lmx1b (non‐Atoh1) are exclusively localized to the rostral KF, while GABAergic FoxP2 neurons are enriched in the caudal KF [81], both with unknown respiratory roles. Excitatory Foxp2 neurons that express Neuropeptide S (Nps) are scattered throughout the PBN with enrichment in the rostral end of the lateral subregion [87]. NPs‐expressing neurons predominantly project via the ventral pathway but also output to medullary regions, including the nucleus tractus solitarius (NTS) [88] and to a lesser extent the VRC [89]. Changes in their activity are associated with sleep‐state transitions, while their stimulation promotes wakefulness and a modest increase in breathing [88]. Thus, this subset of FoxP2 neurons may have a specialized role in coordinating behavioral arousal and breathing, while others maintain breathing stability [76] and contribute to chemoreflexes [86].

The Lmx1b macropopulation comprises at least six transcriptionally distinct subclusters [80], some of which are beginning to be linked to specific respiratory functions. Perhaps best‐studied are Calca‐expressing neurons (encoding CGRP), which are concentrated in the PBel [82] and respond broadly to multi‐modal threats [72, 90]. Roughly half of CO_2_‐activated PBN neurons express Calca, with the other half expressing Foxp2 (see above). Rather than hypercapnic ventilatory responses, Calca neurons mediate CO_2_‐induced arousal [91, 92]. Despite modest influence on basal, homeostatic breathing [91], their activation abruptly suppresses behaviorally linked, non‐homeostatic breathing patterns, such as sniffing [49]—mirroring the suppression of sniffing behavior during CO_2_ exposure [49].

Because Calca neuron activation also induces defensive freezing [72], this suppression of active breathing may reflect a conserved response to perceived danger, prioritizing attention and immobility over exploratory or volitional respiratory behaviors. Whether the same Calca neurons underlie both freezing and breathing regulation remains unclear. Under anesthesia, stimulation of Calca neurons fails to alter breathing [49], suggesting their respiratory effects may be mediated indirectly via forebrain circuits engaged during wakefulness. However, selective activation of ascending Calca circuits does not affect breathing [72]. Retrograde tracing has identified a rostral‐ventral subset of Calca neurons that projects to the hindbrain [82], and transcriptomic analyses reveal two distinct Calca‐expressing PBN clusters [80]. These findings raise the possibility that different Calca subpopulations serve distinct roles: some conveying respiratory sensations such as dyspnea to the forebrain, and others coordinating breathing with threat detection and related affective and behavioral responses.

Tachykinin1 (Tac1), which encodes Substance P, is enriched in three Lmx1b clusters and co‐expressed with Calca in two [80]. Despite this overlap, Tac1 neuron activation elicits distinct behavioral and respiratory effects. Tac1 neurons bias threat responses towards escape rather than freezing [93], and—unlike Calca neurons—their activation potently drives breathing to frequencies not achieved even by stimulation of rhythmogenic preBötC neurons [42, 46], producing a breathing pattern indistinguishable from spontaneous sniffing [49]. These respiratory patterns are tightly coupled to olfaction, startle responses, and affective states [48] and are absent in neonates and develop postnatally, following a distinct developmental trajectory from eupnea [94, 95, 96]. Tac1 neurons send ascending projections to forebrain regions that are partially distinct from Calca neurons [93], and project to the VRC, where selective activation of their terminals is sufficient to reproduce their profound respiratory effects [49]. The opposing behavioral and respiratory roles of Tac1 and Calca neurons have been further clarified by selective activation of Tac1 neurons that do not co‐express Calca (Tac1+;Calca‐). This subset drives even stronger respiratory responses, suggesting that co‐activation with Calca constrains Tac1 output [49]. Tac1+;Calca‐ activation also accounts for most escape‐associated behaviors observed during broader Tac1 activation [93]. The circuit mechanisms behind the opposing interactions of these two glutamatergic populations remain unclear. Strikingly, despite the direct engagement of downstream neurons in the VRC, Tac1+;Calca‐ driven respiratory effects disappear under anesthesia [49], indicating these neurons are strongly gated by state and play a minimal role in homeostatic breathing regulation. Instead, this PBN circuit integrates breathing with active exploratory or escape‐related behaviors—a compelling example of non‐homeostatic, state‐dependent, and behaviorally contingent breathing control (Figure 3C).

Oprm1, which encodes μ‐OR, is a transcriptional marker less useful for distinguishing functional PBN subpopulations but carries high clinical relevance. As discussed, μ‐OR–expressing PBN neurons are key mediators of OIRD, implicating at least some as critical contributors to homeostatic breathing. Inhibiting these neurons suppresses breathing, while activating them enhances it—even under anesthesia [97]. Like many GPCRs, Oprm1 transcripts are often low‐abundance and can fall below detection thresholds in single‐cell RNA sequencing, even with deep coverage (∼100 000 reads/neuron) [80]. However, a few transcripts can sustain tens of thousands of receptors at steady state [80], so transcriptomic data alone [98] can drastically underestimate μ‐OR+ neurons compared to immunohistochemistry [99] or reporter labeling in Oprm1^Cre^‐driver lines [77]. These complementary methods reveal broad μ‐OR expression across the PBN, including Atoh1 and Lmx1b lineages and subtypes such as FoxP2, Calca, and Tac1 [49, 82].

This widespread expression pattern reflects the multimodal respiratory effects of opioids (Figures 2 and 3), which not only depress basal respiratory rate but also impair CO_2_ responses, disrupt behaviorally linked breathing modulation (e.g., sniffing), and alter sleep and arousal [75]. These effects likely reflect actions of Oprm1 neurons in distinct PBN subregions or with different projections. For example, FoxP2+/μ‐OR+ neurons in the KF project to the VRC and provide excitatory input to preBötC neurons that is silenced by opioids [76, 100, 101], though whether this circuit is essential for rhythmogenesis in vivo remains unclear. Other Oprm1 subpopulations in the lateral PBN project to either the VRC or CeA. Though mutually exclusive, both exhibit respiratory‐coupled activity and modulate breathing—potentially coordinated by reciprocal intra‐PBN excitation [97]. These neurons not only influence breathing but offer a therapeutic target: their selective activation—or activation of other excitatory GPCRs they express—can reverse OIRD [102]. Altogether, Oprm1‐expressing PBN neurons span multiple lineages, molecular identities, and output pathways, enabling opioids to act on multiple functional and anatomical nodes in the respiratory network.

Unlike the glutamatergic neurons of the PBN, GABAergic neurons are relatively sparse [103], with modest enrichment in the caudal KF [80]. Their respiratory functions remain underexplored, in part because, unlike excitatory PBN neurons that project to the VRC and spinal cord, GABAergic KF and central lateral PBN (PBlc) neurons project almost exclusively to trigeminal sensory nuclei, with little to no VRC connectivity [85]. This suggests a primary role in vibrissal sensorimotor processing rather than direct respiratory modulation. Yet electrical stimulation of the far lateral or ventral regions of the caudal KF and adjacent trigeminal nuclei can induce hypopnea or apnea [71], hinting that inhibitory PBN outputs may participate in circuits that coordinate respiration with orofacial behaviors. Elucidating these roles will require cell‐type‐ and projection‐specific studies of inhibitory PBN circuits during naturalistic breathing behaviors.

Activation of GABAergic neurons in the PBN suppresses neighboring glutamatergic neurons [104], suggesting local inhibitory circuits. Such local inhibition could underlie the opposing respiratory effects of excitatory subpopulations, such as those expressing Calca and Tac1, and contribute to their strong state‐dependent gating [49]. More broadly, local inhibition may coordinate homeostatic and non‐homeostatic modes of respiratory control. It is intriguing to consider whether Atoh1 and Lmx1b excitatory lineages are inversely coupled by local inhibitory circuits within or across PBN subregions to ensure mutual exclusivity among their diverse respiratory roles. Dissecting these interactions may reveal how emotional and cognitive states override respiratory homeostasis and, when dysregulated or pushed to the extreme, engage ascending circuits that evoke dyspnea.

Inhibitory neurons in adjacent pontine regions also modulate breathing. For instance, GABAergic neurons in the pontine reticular nucleus caudalis (PnC) project to the VRC [105] and are driven by the dorsal anterior cingulate cortex (dACC), forming a cortico‐ponto‐medullary pathway that slows breathing. This circuit is linked to behaviorally driven respiratory changes, such as those during drinking, and likely supports non‐homeostatic control. These PnC GABAergic neurons also send collaterals to anxiety‐related forebrain regions, forming a distributed network that modulates breathing and emotional state in parallel [105]—a possible mechanism underlying the calming effects of slow breathing. Together, these findings underscore the nuanced and underexplored roles of pontine inhibitory neurons in coordinating brainstem respiratory circuits—providing gating, integration, and behavioral flexibility alongside their excitatory counterparts.

## 8 Summary

Breathing is deceptively simple yet biologically complex—both robust and flexible, automatic and volitional, homeostatic and emotional. This review synthesizes recent advances revealing how breathing emerges from multilevel interactions spanning ion channel dynamics, cellular biophysics, local networks, and distributed pontomedullary circuits that adapt rhythm and pattern to changing physiological and behavioral demands. At its core, rhythmogenic neurons in the preBötC have flexible spiking modes tuned by network state and interdependent interactions between intrinsic currents and synaptic inputs. These neurons are embedded in the broader VRC, where coordinated activity, not compartmental modules, shapes inspiratory, post‐inspiratory, and expiratory phases that can reconfigure under stress or sensory input. Beyond the medulla, the dorsolateral pons serves as a bidirectional integrator, linking homeostatic control with emotion, volition, and behavior. The PBN contains transcriptionally and developmentally diverse subpopulations that are functionally specialized: some stabilize homeostatic breathing, while others drive state‐dependent integration with behaviors like sniffing or freezing. This perspective argues against a unitary control mechanism. Rather, breathing emerges as an ensemble computation, built from interdependent substrates whose contributions shift with context. Understanding the full richness of this control system—especially under disease, pharmacological suppression, or emotional dysregulation—will require embracing its complexity, rather than reducing it.

## Author Contributions

N.A.B. conceptualized and wrote the manuscript. E.R., J.S., R.S.P., and J.W.A. contributed to literature review, figure preparation, and editing.

## Data Availability

No new data were generated for this review. All discussed findings are from cited publications.
